# Temperature Responses of Heterotrophic Bacteria in Co-culture With a Red Sea *Synechococcus* Strain

**DOI:** 10.3389/fmicb.2021.612732

**Published:** 2021-05-10

**Authors:** Abbrar Labban, Antonio S. Palacio, Francisca C. García, Ghaida Hadaidi, Mohd I. Ansari, Ángel López-Urrutia, Laura Alonso-Sáez, Pei-Ying Hong, Xosé Anxelu G. Morán

**Affiliations:** ^1^Red Sea Research Center, King Abdullah University of Science and Technology, Thuwal, Saudi Arabia; ^2^Water Desalination and Reuse Center, King Abdullah University of Science and Technology (KAUST), Thuwal, Saudi Arabia; ^3^AZTI, Marine Research, Basque Research and Technology Alliance (BRTA), Sukarrieta, Spain; ^4^Environment and Sustainability Institute, University of Exeter, Penryn, United Kingdom; ^5^Centro Oceanográfico de Gijón/Xixón, Instituto Español de Oceanografía, Gijón, Spain

**Keywords:** *Synechococcus*, heterotrophic bacteria, metabolic ecology, temperature, growth rate, cell size, Red Sea

## Abstract

Interactions between autotrophic and heterotrophic bacteria are fundamental for marine biogeochemical cycling. How global warming will affect the dynamics of these essential microbial players is not fully understood. The aims of this study were to identify the major groups of heterotrophic bacteria present in a *Synechococcus* culture originally isolated from the Red Sea and assess their joint responses to experimental warming within the metabolic ecology framework. A co-culture of *Synechococcus* sp. RS9907 and their associated heterotrophic bacteria, after determining their taxonomic affiliation by 16S rRNA gene sequencing, was acclimated and maintained in the lab at different temperatures (24–34°C). The abundance and cellular properties of *Synechococcus* and the three dominant heterotrophic bacterial groups (pertaining to the genera *Paracoccus*, *Marinobacter*, and *Muricauda*) were monitored by flow cytometry. The activation energy of *Synechococcus*, which grew at 0.94–1.38 d^–1^, was very similar (0.34 ± 0.02 eV) to the value hypothesized by the metabolic theory of ecology (MTE) for autotrophs (0.32 eV), while the values of the three heterotrophic bacteria ranged from 0.16 to 1.15 eV and were negatively correlated with their corresponding specific growth rates (2.38–24.4 d^–1^). The corresponding carrying capacities did not always follow the inverse relationship with temperature predicted by MTE, nor did we observe a consistent response of bacterial cell size and temperature. Our results show that the responses to future ocean warming of autotrophic and heterotrophic bacteria in microbial consortia might not be well described by theoretical universal rules.

## Introduction

Bacterioplankton play significant roles in carbon and nutrient cycling in the oceans (Azam et al., 1983; Jiao et al., 2010; Kirchman, 2016). The interactions between autotrophic and heterotrophic bacteria are essential in marine food webs (Azam and Malfatti, 2007; Buchan et al., 2014), especially in oligotrophic tropical and subtropical regions. There, heterotrophic bacteria consume dissolved organic matter (DOM) ultimately fixed photosynthetically by cyanobacteria (mostly belonging to the genera *Prochlorococcus* and *Synechococcus*) and other small phytoplankton (Azam and Malfatti, 2007). In turn, heterotrophic bacteria facilitate the growth of primary producers including cyanobacteria by supplying fundamental micronutrients including vitamins (Hayashi et al., 2011), amino acids (Christie-Oleza et al., 2015b) or scavenging the reactive oxygen species (ROS) such as hydrogen peroxide (H_2_O_2_) from the environment (Morris et al., 2008, 2011), which can be formed photochemically in the oceans as a result of the photooxidation of DOM (Moffett and Zika, 1987; Moffett and Zajiriou, 1990). H_2_O_2_ can cause cell damage and death in the absence of protective enzymes such as catalase that can decompose it to water and oxygen (Imlay, 2003). Since *Prochlorococcus* lacks this enzyme (Morris et al., 2011) and *Synechococcus* has shown a weak catalase activity (Pena and Bullerjahn, 1995), specific groups of heterotrophic bacteria, by acting as H_2_O_2_ scavengers, can help the growth of both cyanobacteria.

*Synechococcus* is an ancient and genetically diverse genus (Scanlan et al., 2009), partly due to horizontal gene transfer events (Zhaxybayeva et al., 2006; Coutinho et al., 2016). The cell size of *Synechococcus* usually varies between 0.8 and 1.2 μm (Mella-Flores et al., 2012) and its genome size ranges between 2.2 and 2.9 Mb (Scanlan et al., 2009). Based on the pigment composition of phycobiliproteins in the photosynthetic antenna, all *Synechococcus* cells are composed of phycocyanin (PC), whereas some strains contain both PC and phycoerythrin (PE) (Liu et al., 2014), resulting in differently colored strains, ranging from blue-green to pink-orange (Wood et al., 1985; Six et al., 2007). A recent study suggests that marine representatives traditionally known as *Synechococcus* are actually comprised by three distinct taxa, suggesting the new names Ca. *Marinosynechococcus* and Ca. *Juxtasynechococcus* together with *Cyanobium* (Doré et al., 2020). Several *Synechococcus* strains have been found naturally associated with heterotrophic bacteria and are difficult to maintain in axenic conditions (Hayashi et al., 2011; Zheng et al., 2018). Axenic *Synechococcus* and *Prochlorococcus* cultures that were cultivated using antibiotic treatments were able to grow only with a high density of the inoculum (Morris et al., 2008, 2011; Hayashi et al., 2011), but they grew more efficiently when co-cultured with several heterotrophic bacteria (Morris et al., 2008; Hayashi et al., 2011; Zinser, 2018). This adds to the difficulty of obtaining axenic isolates of *Synechococcus* using the common culture isolation method (Zheng et al., 2018). The dominant groups of heterotrophic bacteria associated with different eutrophic and oligotrophic *Synechococcus* strains typically belong to Alphaproteobacteria, Gammaproteobacteria, and Flavobacteria (Zheng et al., 2018), some of the most abundant bacterioplankton classes in the ocean (Du et al., 2006; Willis et al., 2019).

Temperature plays a fundamental role in the physiology of microorganisms (Edwards and Richardson, 2004; Montoya and Raffaelli, 2010). Increasing temperature can impact planktonic microbes either directly by altering their metabolism (Sarmento et al., 2010) and individual size (Daufresne et al., 2009; Morán et al., 2010), or indirectly by shifting their distribution or through increased stratification resulting in reduced nutrient inputs into the upper layers (Behrenfeld et al., 2006; Keeling et al., 2010). Although warming will enhance metabolism in nutrient-sufficient conditions, there is an upper temperature limit that every organism can tolerate (Heinrich, 1977). Traditionally described using the Q_10_ coefficient (Eppley, 1972; Chen and Laws, 2017), another way of approaching the role of temperature in microbial plankton is through the framework of Metabolic Theory of Ecology (MTE) (Brown et al., 2004). Metabolism is obviously the result of a myriad of biochemical reactions (Morowitz et al., 2000; Brown et al., 2004), but some generalizations have been made. According to the MTE, the effect of temperature on metabolic rates such as specific growth rates can be described by the activation energy. For autotrophs, with metabolic rates essentially equaling the mean rate of photosynthetic reactions (Farquhar et al., 1980), an activation energy of ca. 0.32 eV is predicted (Allen et al., 2005; López-Urrutia et al., 2006). For heterotrophs, their metabolic rates approach the mean rate of respiration (del Giorgio and Duarte, 2002; López-Urrutia et al., 2006), resulting in an activation energy of ca. 0.65 eV (Gillooly et al., 2001). Another general ecological theory that has gained recent attention in the marine environment is the so-called temperature-size rule (TSR), which suggests an inverse relationship between ambient temperature and individual size (Atkinson, 1994; Atkinson and Sibly, 1997). Originally developed for metazoans (Azevedo et al., 2002), the decrease in individual size as a function of temperature has also been documented for picophytoplankton (Morán et al., 2010) and heterotrophic bacteria (Morán et al., 2015).

Global warming is impacting the structure and function of marine ecosystems worldwide. Although tropical planktonic bacteria encounter the highest natural temperatures in the world, there is no systematic information about how they cope with them or their potential adaptation to further warming. Understanding the physiological responses of a Red Sea *Synechococcus* strain and their associated heterotrophic bacteria to temperature will provide us with an estimation of how future warming may impact these crucial players in biogeochemical cycling in regions currently subject to lower temperature ranges. In this study, we used the non-axenic *Synechococcus* RS9907 culture previously acclimated to different temperatures spanning from 24 to 34∘C. The strain RS9907 was originally isolated from the Gulf of Aqaba (29∘28′N, 34∘55′E) in the Red Sea (Fuller et al., 2003) and belongs to the clade II, the dominant in the Red Sea (Post et al., 2011; Coello-Camba et al., 2020). Although kept in culture for thousands of generations, disentangling how these Red Sea bacteria cope with high temperatures can help us predict future responses of bacterioplankton in other marine regions.

## Materials and Methods

### Culture Conditions

The *Synechococcus* sp. RS9907 culture was obtained from the Roscoff Culture Collection (Roscoff, France), and grown in artificial seawater (PCR-S11-Red Sea medium prepared with a salinity value of 36) at 22∘C under an irradiance of ca. 115 μmol photons m^–2^ S^–1^ in a 12:12 h light:dark cycle until the start of the experiments. Although according to Doré et al. (2020) we should rename it to *Marinosynechococcus*, we have opted to keep the traditional term until a wide consensus about renaming the group is reached.

### Thermal Acclimation and Flow Cytometry

After acclimating the culture to the different temperatures for at least 8 generations as described in Palacio et al. (2020), 3 experiments were sequentially conducted targeting different groups and growth phases of the bacterial consortium (Supplementary Table 1). Co-culture true replicates were incubated in 160 mL vented cap flasks at 4–5 different temperatures ranging from 24 to 34∘C in order to determine their thermal niches (Boyd et al., 2013). The first experiment (Experiment A), focused on monitoring the dynamics of RS9907 rather than the heterotrophs, and the cultures were incubated at 24, 28, 30, and 33∘C. The sampling was done daily during the light cycle at 9:30 a.m. for a variable total duration depending on the actual experimental temperature (i.e., the warmer the shortest duration). Four replicates were sampled until *Synechococcus* entered the logarithmic growth phase (after 4–5 days) and from then onward separate duplicates were kept and monitored until the stationary phase was reached (7–13 days), in order to accurately estimate the carrying capacity (i.e., maximum abundance) of *Synechococcus*. The second experiment (Experiment B) lasted for 4 days and consisted of triplicates incubated at 24, 28, 30, and 33°C. No sampling was done in the first 2 days and samples were taken every 2 h from day 2 to 4 to finely document the exponential growth phase of the co-culture. In the third experiment (Experiment C), specifically aimed at targeting the dynamics of heterotrophic bacteria, In addition to the tested temperatures in experiments A and B, one set of triplicates was incubated at 34∘C. Samples were collected every 2 h for 24 h and one more sample was taken after 48 h. During the acclimatization process, it became clear that any temperature above 30∘C should be done with caution to avoid killing the cyanobacteria. We tried to determine the maximum temperature at which *Synechococcus* could grow. Since this study is more focused on experiment C, we acclimated the cultures at a temperature slightly higher than 33∘C but still within the Red Sea’s natural temperature range.

All samples (396 μl) were preserved in cryovials with glutaraldehyde (0.025% final concentration), incubated in the dark for 10 min to allow the cells to be fixed completely and then stored at –80∘C until analysis (Palacio et al., 2020). Typically within 1 week from collection, samples were run using a BD FACSCanto II flow cytometer and the obtained data were analyzed using BD Paint-A-Gate software. The *Synechococcus* cells were easily detected in cytograms of red fluorescence (PerCP-Cy5-5, 498 nm) vs. right angle light or side scatter (SSC) signals and of red vs. orange fluorescence (PE, 433 nm) signals. After staining the samples with SYBRGreen (Marie et al., 1999), heterotrophic bacteria were identified and counted simultaneously using cytograms of green fluorescence (FITC, 360 nm) vs. red fluorescence signals in order to distinguish between autotrophs (naturally fluorescing in red) and heterotrophs, and also of green fluorescence vs. SSC signals. SSC is a flow cytometric variable common to all cells reflecting changes in individual size (Felip et al., 2007). The mean relative SSC (relSSC) values of the different flow cytometric groups to the SSC value of 1 μm fluorescent latex beads (Molecular Probes) were used to estimate their corresponding cell diameter (μm) using these calibrations: μm = 1.62 + 0.87 log relSSC for *Synechococcus* and μm = 0.91 + 0.34 log relSSC for the heterotrophic groups (Calvo-Díaz and Morán, 2006). Bacterial cell size was finally given as biovolume in μm^3^ assuming spherical shape. We are aware that some of the bacteria present may well have been bacilli (rods) rather than cocci (spheres). Although the co-culture was not observed under the microscope to detect the exact cell shapes, since the analysis by flow cytometry is done cell by cell to a large total number (ca. 10,000 cells), it can be presumed that the relSSC value of a bacillus with the same width of a coccus’ diameter (but a clearly longer length), would ultimately be higher.

### DNA Extraction and 16S rRNA Gene Amplicon Sequencing

All DNA samples in Experiment C were collected after 48 h, a time at which the abundance of heterotrophic bacteria was higher than that of RS9907 at all tested temperatures (Supplementary Table 2). 130 mL of the *Synechococcus* RS9907 co-cultures in the available flasks were collected after 48 h, divided into three equal volumes and centrifuged at 4,000 rpm for 20 min at 4∘C. The supernatant was removed and 15 mL was kept from each tube and mixed together in one tube. The 45 mL of mixed concentrated culture was centrifuged again at 4,000 rpm for 20 min at 4∘C. The supernatant was removed and pellets were re-suspended and transferred to 2 mL tube. Samples were centrifuged at 14,000 rpm for 2 min at 4∘C and the supernatant was discarded. DNA pellets were stored at –80∘C until DNA extraction. DNA was extracted using DNeasy PowerSoil kit (QIAGEN, Germany) with a minor modification to the protocol by adding 15 μL of 100 mg/mL lysozyme to the pellets and incubation at 37∘C (Ansari et al., 2015). PCR amplification of the variable regions 4 and 5 of the 16S rRNA gene was performed using the forward primer 515F-Y (5′-GTGYCAGCMGCCGCGGTAA-3′) and reverse primer 926R (5′- CCGYCAATTYMTTTRAGTTT-3′) (Parada et al., 2015). PCR reaction mixture was prepared for all samples with 0.6 μl of each primer, 0.48 μl of Taq DNA polymerase HiFi (Quanta, BioSciences, Inc.^TM^), and 15 μl of FailSafe^TM^ PCR 2X PreMix (Epicenter, WI, United States) in a total volume of 30 μl. Triplicate of 10 μl PCRs were performed with 25 cycles of thermal program following cycling conditions of 926R primer (Parada et al., 2015). These triplicate samples were then pooled, and 2 μl was used on a 1% agarose gel to check the amplification. The remaining 28 μl was cleaned up using AMPure XP beads (Beckman Coulter). Indexing PCR was performed on the cleaned samples using Nextera XT index kit (Illumina, San Diego, CA, United States) following the manufacturer’s protocol. The MiSeq spacer size was 412 bp and 16S rRNA gene library was sequenced using Illumina MiSeq platform in 2 × 300 bp paired-end mode with 7x depth of coverage. A 20% phiX was used as a control at the KAUST Bioscience Core Laboratory.

### Sequence Data Processing and Analysis

Raw sequence data analysis was conducted in mothur (version 1.39.5) following Hadaidi et al. (2018) with some modifications. Sequence reads were joined into contigs using the “make.contigs” command, and contigs longer than 413 bp and ambiguous bases were removed from the analysis. All the sequences that occurred only once were subsequently excluded and SILVA database was used to align the remaining sequences. Sequences were then pre-clustered letting maximum 2 nt difference between the sequences and chimeras were removed applying “uchime.” The sequences were assigned to Operational Taxonomic Units (OTUs) using the Greengenes database. Short-sequence reads did not allow the identification of species, hence our analysis was conducted at the genus level.

In order to explore the correspondence between the cytometric and taxonomic groups present in the co-culture, the abundance of the dominant taxa was estimated by multiplying their relative abundance in the amplicon sequencing dataset by the flow cytometry total bacterial abundance. The use of amplicon sequencing relative abundance multiplied by the abundance obtained by flow cytometry to provide an estimate of the abundance of the different OTUs is rather common (Props et al., 2017; Lin et al., 2019). Although prone to amplification biases, if only a few groups clearly stand out as the most abundant ones (as found in our co-culture), the potential error would be minimized. Since *Synechococcus* was unequivocally identified in the cytograms, its flow cytometric abundance was used as a control to match that based on percent reads.

### Metabolic Theory of Ecology (MTE) and Temperature-Size Rule (TSR) Calculations

To estimate the specific growth rates (μ, d^–1^) of *Synechococcus* and the heterotrophic bacterial groups distinguished by flow cytometry at the different temperatures of each of the three experiments, we determined the slope of ln-transformed abundance vs. time for the corresponding exponential phase of growth (Huete-Stauffer et al., 2015), which differed in timing for the different groups and experimental temperatures. The temperature responses of bacterial specific growth rates and carrying capacities (i.e., maximum abundances) were approached using the MTE central equation (Brown et al., 2004). Specifically, the μ thermal dependence was described by its activation energy (*E*_*a*_). *E*_a_ was calculated in Arrhenius plots as the slope of ln-transformed specific growth rates vs. 1/kT (Huete-Stauffer et al., 2015), with k representing the Boltzmann’s constant (8.62 × 10^–5^ eV K^–1^), and T the temperature in Kelvin (Brown et al., 2004). The carrying capacity of each bacterial group was recorded as the maximum abundance that was actually reached at a given incubation temperature. In order to test the MTE that predicted inverse relationship between carrying capacity and temperature (Savage et al., 2004), we simply calculated the linear regression between both variables. The same method was used for testing the prediction of the TSR of a smaller individual size with higher temperature (Atkinson et al., 2003; Bernhardt et al., 2018). We represented the mean bacterial biovolume (see calculation details in the above section) over the exponential growth phase vs. experimental temperature and calculated the corresponding slope of the linear regression model.

## Results

### Identification of *Synechococcus* sp. RS9907-Associated Heterotrophic Bacteria

**FIGURE 1:**
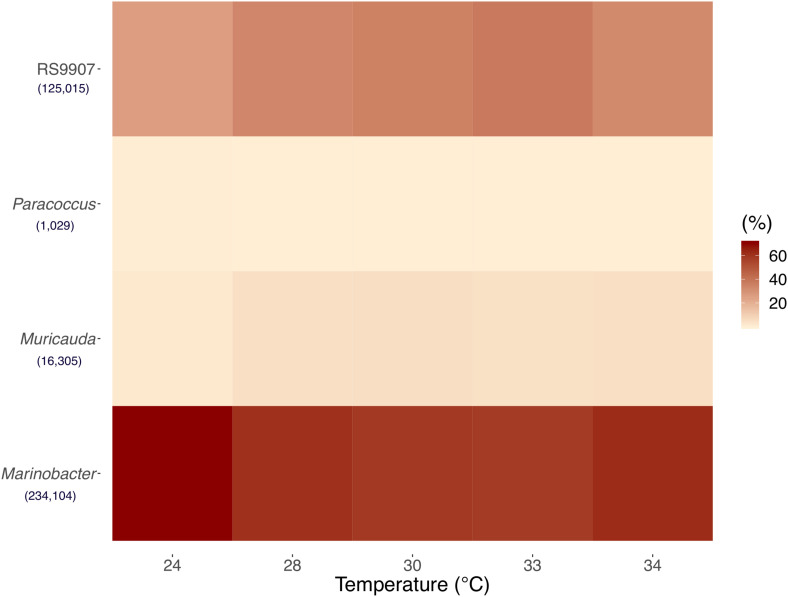
Mean of triplicates 16S rRNA gene-based relative abundances of *Synechococcus* and the top three most abundant heterotrophic bacteria OTUs at the five incubation temperatures of Experiment C after 48 h. See the text for details. Numbers in brackets represent total OTUs reads.

To identify the heterotrophic bacteria associated to *Synechococcus* in the RS9907 co-culture, we analyzed 16S rRNA gene amplicon sequences. A total of 1396590 sequences with an average read length of 412 bp reads were obtained by amplicon sequencing targeting the 16S rRNA in the co-cultures acclimated at the five different temperatures (24, 28, 30, 33, and 34∘C) after 48 h of incubation in experiment C (see above for details about the three experiments performed). After passing the quality filtering and exclusion of chimeras, 837308 sequences were retained. Sequences were then rarefied to 25,188 sequences per sample and their taxonomic affiliation was analyzed. At a 97% similarity cutoff, sequences were clustered into 49 Operational Taxonomic Units (OTUs). The three OTUs that had the highest relative abundances, excluding RS9907 (33.09% of the total) were affiliated to the genera *Marinobacter* (Gammaproteobacteria), *Muricauda* (Flavobacteriia), and *Paracoccus* (Alphaproteobacteria) (Figure 1). *Marinobacter* had a mean relative abundance of 61.96%, followed by *Muricauda* (4.32%), and *Paracoccus* (0.27%) (Figure 1), while the sum of the remaining 49 OTUs identified amounted to only 0.4% of the reads. The fifth most relatively abundant OTU (not shown) represented on average 0.2% of the reads. Even though the field is moving toward using amplicon sequence variants (ASV)-based sequencing analysis, using ASVs in a co-culture that did not contain a very diverse community and aiming at genus-level identification would not have been very different from our reported OTUs. According to Martinson et al. (2019), both OTU- and ASV-based analysis methods would likely have yielded nearly identical results in our study.

**FIGURE 2:**
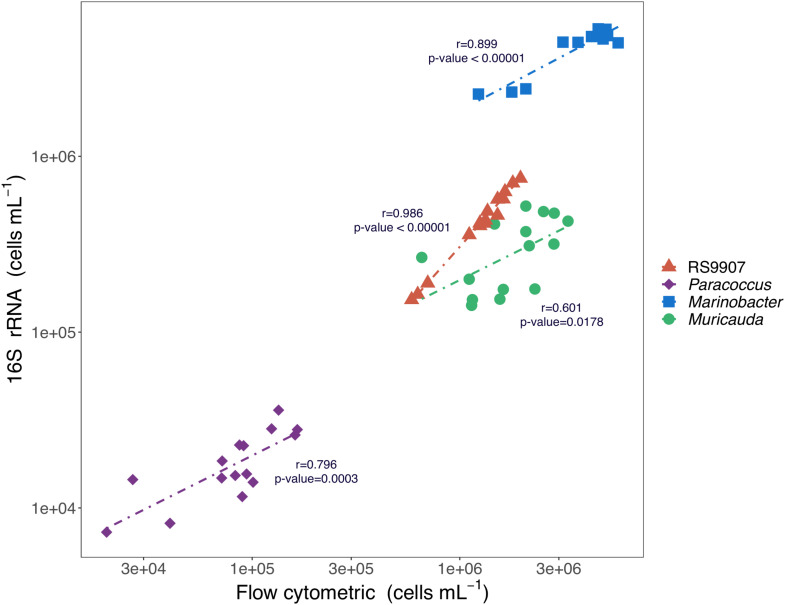
Relationships between the 16S rRNA gene-based cell abundances (i.e., percent reads multiplied by total bacterial abundance as determined by flow cytometry) of *Synechococcus* RS9907, *Paracoccus*, *Marinobacter*, and *Muricauda* and the flow cytometric abundances of *Synechococcus* and the 3 major clusters of heterotrophic cells shown in Supplementary Figure 1 for all replicates and temperatures after 48 h incubation of experiment C.

Three distinct heterotrophic bacterial groups were also consistently detected in the cytograms from the three experiments based on their relative nucleic acid content and cell size characteristics. The groups were initially named as 1, 2, and 3 ordered by decreasing values of green fluorescence and SSC (Supplementary Figure 1). After comparing their flow cytometry-based abundances (pooling all replicates and temperatures) together with that of RS9907 (easily distinguished because of its higher red fluorescence signal due to chlorophyll *a*) with the 16S rRNA gene-based abundance of the 4 dominant OTUs, we found significant correlations with *r*-values ranging from 0.6 to 0.9 over 3 orders of magnitude absolute abundances. Hereinafter, the flow cytometric groups 1, 2, and 3 will be referred to as *Paracoccus*, *Marinobacter*, and *Muricauda*, respectively (Figure 2).

### Bacterial Dynamics and Cell Size

**FIGURE 3:**
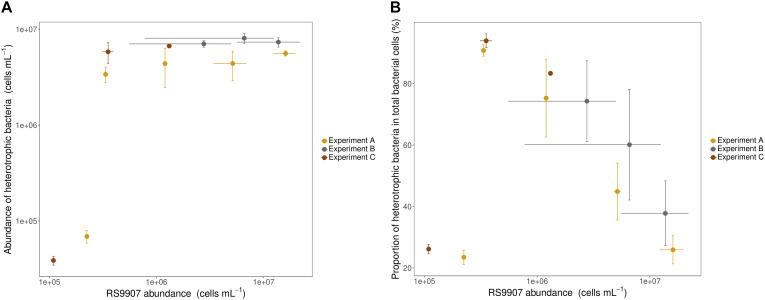
**(A)** Absolute abundance of heterotrophic bacteria (i.e., sum of all heterotrophic cells counted by flow cytometry) and **(B)** proportion of heterotrophic bacteria to total bacterial cells vs. the abundance of RS9907 at 28°C in the three experiments. Each symbol corresponds to one daily measurement. Error bars represent the standard deviations of 4 replicates in experiment A and triplicates in experiments B and C.

To document the dynamics of the RS9907 co-culture, three experiments were conducted at different bacterial growth phases and time points. Total heterotrophic bacterial abundance was slightly lower than that of *Synechococcus* at the onset of experiments A and C, with cyanobacteria ranging from 1.1 to 2.2 × 10^5^ cells mL^–1^. However, the total abundance of heterotrophic bacteria increased very fast (just after 1 day of incubation) and remained relatively constant at 4–7 × 10^6^ cells mL^–1^ while *Synechococcus* increased in abundance over two orders of magnitude (Figure 3A). Consequently, the proportion of heterotrophic bacteria in the co-culture decreased steadily and similarly in the 3 experiments from ca. 90 to ca. 25% as the abundance of RS9907 increased to a maximum of ca. 1.6 × 10^7^ cells mL^–1^ (Figure 3B). By plotting data from the three experiments corresponding to different stages in the RS9907 co-culture dynamics, it became clear that the importance of heterotrophic bacteria decreased with increasing abundance of *Synechococcus* once the cyanobacteria reached ca. 3.5 × 10^5^ cells mL^–1^.

**FIGURE 4:**
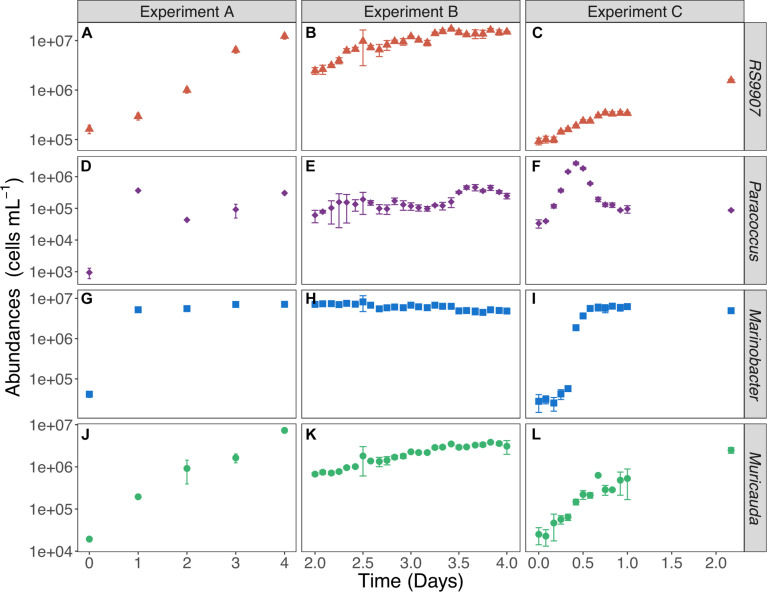
Variations in the mean abundance of *Synechococcus* RS9907 **(A–C)** and the 3 heterotrophic bacteria [*Paracoccus* **(D–F)**, *Marinobacter* **(G–I)**, *Muricauda* **(J–L)**] at 30°C in the three experiments. Error bars represent the standard deviations of 4 replicates in experiment A and triplicates in experiments B and C.

The three experiments allowed us to detect highly variable but internally consistent responses of the three most abundant heterotrophic bacterial OTUs, with a different degree of temporal resolution (Figure 4). Experiment A and C were performed with lower initial abundance of both autotrophic and heterotrophic cells than experiment B (Supplementary Table 1). Experiment A began with low abundance (1.6 × 10^5^ ± 32538 cells mL^–1^) of *Synechococcus* (Figure 4A), experiment B started with higher initial abundances (2.5 × 10^6^ ± 384751 cells mL^–1^, Figure 4B) while experiment C was purportedly conducted with a lower initial abundance (9.2 × 10^4^ ± 14,382 cells mL^–1^, Figure 4C), in order to better track the fine-scale dynamics of heterotrophic bacteria. Although the initial abundance of RS9907 in experiment A (Figure 4A) and experiment C (Figure 4C) were closer, the daily sampling of experiment A did not allow us to adequately determine the response of the three heterotrophic OTUs. Experiment C was indeed the best suited to document the actual responses of the heterotrophic bacteria. In particular, *Paracoccus* showed very distinct dynamics compared to the other two groups: it invariably responded with a fast increase and decrease in the first 24 h (Figures 4D–F) at all temperatures (Supplementary Figures 2D–F, 3D–F, 4D–F, 5B). *Paracoccus* reached their maximum abundance after 1 day in experiment A, which was comparable to the abundance after 24 h in experiment C. Frequent sampling during the first 24 h in experiment C provided us with not only an assessment of the specific growth rate, but also a better estimation of the maximum abundance of *Paracoccus*. *Marinobacter* reached their carrying capacity of ca. 10^7^ cells mL^–1^ after <1 day in experiments A and C and remained pretty constant hereinafter (Figures 4G–I) at all temperatures (Supplementary Figures 2G–I, 3G–I, 4G–I, 5C). Similarly, the every 2 h regular sampling of experiment C allowed us to measure the specific growth rate of *Marinobacter*. In contrast, *Muricauda* kept on growing during the sampled periods in the three experiments, following a response similar to that of *Synechococcus*, though with lower abundance (Figures 4A–C,J–L) at all temperatures (Supplementary Figures 2A–C,J–L, 3A–C,J–L, 4A–C,J–L, 5A,D). All other tested temperatures showed similar patterns to the bacterial dynamics found at 30∘C (Supplementary Figures 2–5).

The differences in mean cell size of the 4 bacterial OTUs were almost consistent across the three experiments (Supplementary Table 3). Expectedly, *Synechococcus* were the largest bacteria, followed by *Paracoccus* and *Marinobacter*, while *Muricauda* showing the smallest cell size. The effects of temperature on the cell size of each group are discussed in the following section.

### Temperature Responses

**FIGURE 5:**
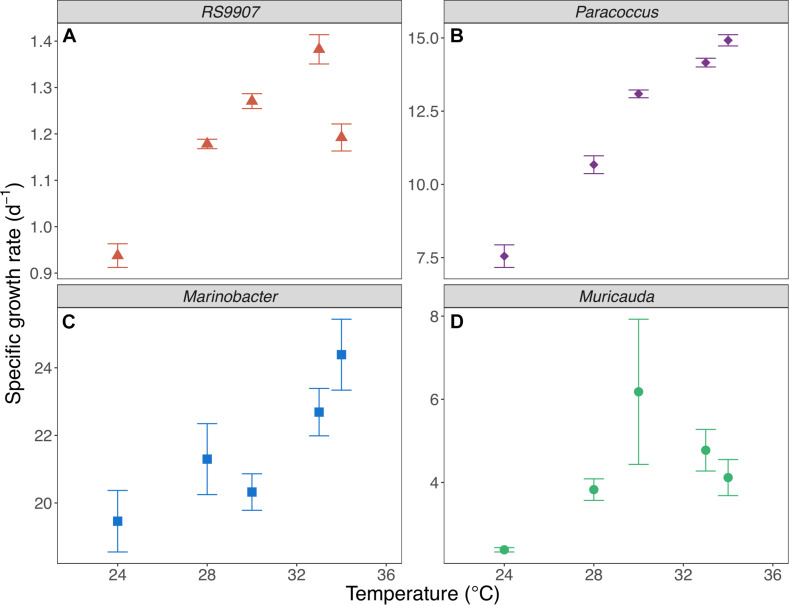
Mean specific growth rates of *Synechococcus* RS9907 **(A)**, *Paracoccus* **(B)**, *Marinobacter* **(C)** and *Muricauda* **(D)** vs. incubation temperature in experiment C. Error bars represent standard deviations.

**FIGURE 6:**
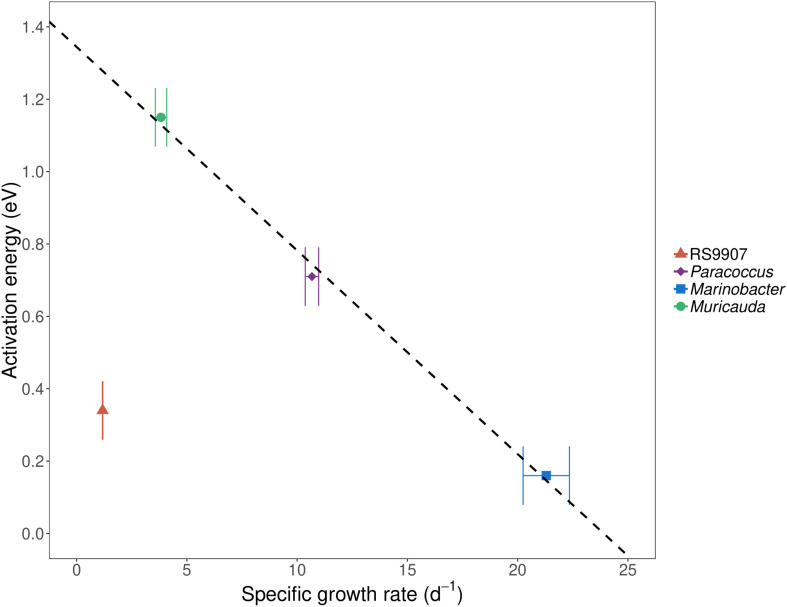
Mean activation energy (*E*_a_) estimated from specific growth rates for at least three different temperatures (see section Materials and Methods and Figure 5 for details) vs. mean specific growth rate (μ) at 28°C of *Synechococcus* RS9907, *Paracoccus*, *Marinobacter*, and *Muricauda* in experiment C. Error bars represent standard deviations. The fitted line represents the ordinary least squares linear regression for heterotrophs (*r*^2^ = 0.99, *p* = 0.04, *n* = 3). The activation energy of *Marinobacter* was estimated from specific growth rates at all tested temperatures (24–34°C) while *Muricauda* and *Paracoccus* were estimated including only temperatures from 24 to 30°C. For *Synechococcus*, the activation energy was calculated from specific growth rates from 24 to 33°C.

To determine the joint responses of the autotrophic and heterotrophic members of the co-culture to experimental warming using the MTE and TSR frameworks, we assessed the specific growth rates and cell sizes of the dominant members of the co-culture over a temperature range covering the natural variability found in Red Sea waters (24–34∘C). In particular we wanted to test these hypotheses that (i) the specific growth rates of autotrophic and heterotrophic bacteria will have different temperature dependences (i.e., they differ in their corresponding MTE activation energies) and (ii) the cell size will decrease with warming in a similar fashion for all the bacterial groups identified. *Marinobacter* showed the highest specific growth rates of the four bacterial OTUs assessed in experiment C (Figure 5), with values exceeding 20 d^–1^ (19.6–24.4 d^–1^), followed by *Paracoccus* (7.60–14.9 d^–1^) and *Muricauda* (2.38–6.18 d^–1^). The specific growth rates of the RS9907 strain (0.94–1.38 d^–1^) were much lower than those of the three heterotrophs. Specific growth rates of all groups increased with temperature albeit with different patterns, resulting in different activation energies (see below). RS9907 showed the highest specific growth rates at 33°C, *Paracoccus* and *Marinobacter* grew best at 34∘C and *Muricauda* showed a decrease in their specific growth rate at temperatures higher than 30∘C (Figure 5). The specific growth rate of *Synechococcus* in experiment A (1.00–1.60 d^–1^) was very similar to the values shown for experiment C and also followed a similar pattern with temperature. The corresponding activation energy (*E*_a_) of RS9907 (0.34 ± 0.02 eV) was not significantly different from the value hypothesized for autotrophs by the MTE (0.32 eV, *t*-test, *p* > 0.05) while the *E*_a_ values of the three heterotrophs varied greatly, from 0.16 to 1.15 eV. Interestingly, the activation energies of *Paracoccus*, *Marinobacter*, and *Muricauda* were inversely correlated with their mean specific growth rates (Figure 6).

The decay phase of *Paracoccus* came just after a few hours of incubation during the first 24 h (the maximum abundance was reached ca. half a day after the start of the incubation), allowing us to estimate an apparent mortality rate, which ranged from –1.98 to –12.01 d^–1^. Similar to specific growth rates (Figure 5B), mean mortality rates were significantly and negatively correlated with increasing temperature in the 5 temperatures tested of experiment C (*r*^2^ = –0.89, *p* = 0.001, *n* = 5, Supplementary Figure 6). Consequently, both mean specific growth and mortality rates of this group showed a strong correlation across the temperatures tested (*r*^2^ = –0.96, *p* = 0.001, *n* = 5).

The carrying capacities (i.e., maximum abundances) of *Muricauda* and *Synechococcus* could only be accurately estimated from experiment A, since they both kept on growing in experiment C. The latter experiment permitted the determination of the carrying capacities of *Paracoccus* and *Marinobacter*. The carrying capacities of *Synechococcus* (2.94 × 10^7^–1.17 × 10^8^ cells mL^–1^) and *Paracoccus* (1.86–4.01 × 10^6^ cells mL^–1^) were negatively correlated with temperature (*r*^2^ = –0.69 and –0.41, *p* = 0.007 and 0.006, *n* = 8 and 15, respectively). On the contrary, a positive correlation was observed between *Marinobacter* carrying capacity (4.89–7.03 × 10^6^ cells mL^–1^) and temperature (*r*^2^ = 0.56, *p* = 0.003, *n* = 12, excluding 34∘C values). The carrying capacities of *Muricauda* (3.16–7.32 × 10^6^ cells mL^–1^) did not show any significant correlation with temperature (Supplementary Figure 7A).

The TSR was tested for the different bacterial groups with the mean cell size during the exponential growth phase. Interestingly, there was a strong positive relationship between the mean cell size of RS9907 during the exponential growth phase (0.39–0.60 μm^3^) and temperature (*r*^2^ = 0.88, *p* = 0.0001, *n* = 9, excluding > 30∘C values), while none of the heterotrophic groups mean cell sizes bore any significant correlation with temperature (Supplementary Figure 8). However, we also estimated the corresponding cell size at maximum abundance of the 4 OTUs at each experimental temperature. *Marinobacter* cell size was the only bacterial group significantly correlated with temperature (*r*^2^ = 0.59, *p* = 0.0005, *n* = 15), similarly to the result of RS9907 (Supplementary Figure 7B). The effect of temperature was consistent on the mean cell sizes across the three experiments (Supplementary Table 3). Mean cell sizes of *Synechococcus* also increased with temperature in experiments A, B, and C (*r*^2^ = 0.98; 0.88; 0.59, *p* < 0.00001; < 0.00001; = 0.005, *n* = 16; 12; 15, respectively) with slightly different values while heterotrophic bacteria did not show any significant relationship with temperature.

## Discussion

### Heterotrophic Bacteria in the *Synechococcus* sp. RS9907 Co-culture

The heterotrophic bacterial OTUs present in the RS9907 co-culture, making up to a mean of 66.69% of total reads when *Synechococcus* reached ca. 1 million cells mL^–1^ mostly belonged to Gammaproteobacteria and Alphaproteobacteria. Coincidentally, they are also the most abundant classes of heterotrophic bacterioplankton in the Red Sea, with Flavobacteriia typically found at lower abundances (Ngugi et al., 2012; Pearman et al., 2016, 2017), and only increasing in their importance in shallow waters (Ansari, personal communication), suggesting that their presence in the non-axenic culture dates back from the time of initial isolation.

The flow cytometric abundance of the 4 clusters we consistently observed (including *Synechococcus*) aligned independently with the read-based abundance of the top four dominant bacterial OTUs (Figure 2). Since these abundances spanned over three orders of magnitude, we can conclude that the affiliation of the top three most abundant heterotrophic bacterial OTUs to the genera *Marinobacter*, *Muricauda* and *Paracoccus* overcome any possible amplification bias of the 16S rRNA gene. *Marinobacter* spp. have been previously associated with dinoflagellate and coccolithophore (Amin et al., 2009) as well as diatom cultures (Amin et al., 2009, 2015). Several *Marinobacter* species have been reported to effectively promote the growth of different dinoflagellate strains (Amin et al., 2009; Bolch et al., 2017), partially due to the production of vibrioferrin, a siderophore secreted by the bacteria that stimulates algal absorption of iron (Amin et al., 2009). In return, not only in the decay phase but during exponential growth, phytoplankton naturally release DOM (Myklestad, 1995; Hansell, 2013), which can be utilized by the heterotrophic bacteria in the surrounding water or within the phycosphere, suggesting that this essential iron and fixed carbon mutualistic interaction may also occur between the *Marinobacter* species present in the RS9907 co-culture. Likewise, different *Muricauda* strains have also been found to promote the growth of microalgae, whose increase in cell density was accompanied by an increase in *Muricauda* cells number (Han et al., 2016). A strain of *Muricauda* was shown to contain appendages, vesicle-like structures with fibrillar-like structures on the surface, which are thought to be utilized to connect cells to one another (Bruns et al., 2001) and perhaps involved in interactions with the surrounding environment (Malfatti and Azam, 2009). Those might account for the positive correlation between the growth patterns of our *Muricauda* OTU and *Synechococcus.* Finally, a strain of *Paracoccus* has shown positive oxidase and catalase activities (Zheng et al., 2011) and a reduction in H_2_O_2_ has been reported in different *Prochlorococcus* strains when they were co-cultured with heterotrophic bacteria (Morris et al., 2008, 2011; Coe et al., 2016). Our *Paracoccus* member may play a role in scavenging the ROS possibly generated in the co-culture, since *Synechococcus* lacked its own catalase when we blasted the katG gene against the complete genome of the RS9907 strain. Remarkably, the same three genera of heterotrophic bacteria reported here were also found in *Synechococcus* sp. BL107 (Christie-Oleza et al., 2017), originally isolated from Blanes Bay, Mediterranean Sea (Dufresne et al., 2008). The heterotrophic bacterium presented in the WH7803 co-culture was found to rely on the exoproteome of the cyanobacterium, as the shotgun proteomics analysis showed that there were virtually no accumulated proteins in this co-culture (Christie-Oleza et al., 2015a). In contrast, the reduction of the accumulated organic matter generated by *Synechococcus* by one of the heterotrophic bacteria present was found to decelerate cyanobacteria mortality in the above-mentioned study (Christie-Oleza et al., 2017), suggesting that although no *Roseobacter* member was found among our dominant OTUs, this behavior might contribute to the apparent symbiotic relationship established between *Synechococcus* and the heterotrophic bacteria.

### Bacterial Dynamics and Cell Size in Response to Temperature

Although when applied to prokaryotic plankton neither the MTE (Sinsabaugh and Shah, 2010; Huete-Stauffer et al., 2015; Arandia-Gorostidi et al., 2017; Morán et al., 2018) nor the TSR (Morán et al., 2015) are free from criticism, they are still valid frameworks to test general relationships of organisms in response to temperature. We have used both the MTE and TSR principles in the controlled conditions of a co-culture in which the cyanobacterium and likely also the associated heterotrophs had been isolated from the Red Sea, one of the warmest marine basins on earth. According to MTE, the response to temperature can be assessed by the so-called activation energy (*E*_a_). Simply put, the higher their *E*_a_, the higher their temperature dependence of μ. In theory, in nutrient-sufficient conditions, temperature increases biochemical reaction rates exponentially, resulting in a similar exponential dependence for the integrated growth rates of autotrophs on one side and heterotrophs on the other one (Brown et al., 2004). Although μ steadily increased with warming in all four bacterial groups (Figure 5), the *E*_a_ values for heterotrophs were not fully consistent. The mean activation energy of *Synechococcus* in experiment C (very similar to the other two experiments estimates) was 0.34 eV ± 0.02, statistically indistinguishable from the value hypothesized for autotrophs by the MTE (0.32 eV). However, the μ *E*_a_ values for the three top most abundant heterotrophic bacteria OTUs (making on average 2/3 of the total bacterial counts) were notably more variable, resulting in a huge variation in the specific growth rate response to temperature (Figure 5). Although their mean activation energy (0.67 eV) also virtually equaled the corresponding MTE value for heterotrophs (0.65 eV), this value resulted from a similar *E*_a_ in the case of *Paracoccus* (0.71 ± 0.04 eV) but higher for *Muricauda* (1.15 ± 0.20 eV) and much lower value for *Marinobacter* (0.16 ± 0.03 eV). These values indicate that *Muricauda* μ was most affected by temperature while *Marinobacter* was the least. The negative relationship between the specific growth rates of the three heterotrophic bacterial groups (we chose to represent 28°C as an example, but it held for all other experimental temperatures) and their estimated activation energies was puzzling (Figure 6). One could argue that for temperature to have the predicted effect (i.e., *E*_a_ = 0.32 eV for *Synechococcus* and 0.65 eV for the heterotrophs), tropical bacteria should not be neither bottom-up nor top-down controlled (Morán et al., 2018). Theoretically, in the absence of predators, autotrophic and heterotrophic bacterial needs for carbon and all other nutrients required for growth should be met by the culture medium. It may have not been the case for *Marinobacter*, either because some key nutrient or vitamin was in short supply although its *μ*-values were very high. Indeed, the specific growth rates of the three heterotrophic bacteria assessed here were however much higher than the bulk values measured in coastal Red Sea waters, even for the presumably copiotrophic high nucleic acid (HNA) content group of cells (1.01–2.33 d^–1^, Silva et al., 2018), suggesting that not only nutrient conditions affects *E*_a_ values (Arandia-Gorostidi et al., 2017; Morán et al., 2018), but that the temperature dependence is also species-specific and related to the identity of the heterotrophic bacteria living in close association with the same cyanobacterium.

An increase in temperature within the normal, physiological range or thermal niche will increase population growth rate when resources are abundant and densities are low (Brown et al., 2004; Sherman et al., 2016), while increasing temperature is hypothesized to decrease the carrying capacity (Brown et al., 2004; Bernhardt et al., 2018). The MTE thus predicts an inverse relationship between carrying capacity and temperature, due to the fact that higher temperature will raise the metabolic rate, so that each individual will use resources at a higher rate (Savage et al., 2004). However, our results showed that each bacterial group responded differently in terms of the temperature response of their carrying capacity. The negative effect of temperature on the maximum abundance of *Synechococcus* and *Paracoccus* followed perfectly the MTE prediction whereas *Marinobacter* showed a significant yet opposite trend (Supplementary Figure 7A). In previous studies with mixed heterotrophic bacterial assemblages in temperate waters, the carrying capacity was shown to increase rather than decrease with temperature, an observation linked to the nutrient availability along the seasonal cycle (Huete-Stauffer et al., 2015; Arandia-Gorostidi et al., 2017). Similar to the specific growth rates discussed above, species-specific responses appear as critical, preventing the lack of a coherent response shown by the three heterotrophic bacteria that dominated the RS9907 co-culture, even if we assumed nutrient-sufficient conditions.

The temperature-size rule (TSR, Savage et al., 2004) is another general ecological theory that only relatively recently has been tested with marine planktonic organisms (Daufresne et al., 2009; Morán et al., 2010, 2015; Forster et al., 2012; Huete-Stauffer et al., 2016). Cell size reduction could be an acclimation response to conditions that imply increasing population growth rate, and to compensate for the increasing demand for resources (Atkinson et al., 2003). It is generally thought that resources can be obtained more effectively in smaller cells, hence facilitating their growth with increasing temperatures (Atkinson and Sibly, 1997). An exception to the TSR may arise if resource limitations are removed or reduced as a consequence of warming (Atkinson et al., 2003). Remarkably, although a trend toward generalized larger sizes in the exponential phase with higher temperature was shown also by the three major heterotrophic bacteria OTUs (Supplementary Figure 8), *Synechococcus* was the only species in our co-culture showing a consistent, highly significant variation of bacterial cell size with temperature, but it was exactly the opposite of the TSR prediction. Similar to our study, a strong positive correlation was found between *in situ* temperature and the mean cell size of a natural population of *Synechococcus* characterized by low PE fluorescence in shallow Red Sea waters (Supplementary Figure 9). A recent study with data from the experiment labeled as A in this study provided an alternative explanation for the fact that RS9907 did not show the expected negative relationship between cell size and temperature at any time point during the diel cycle. Palacio et al. (2020) however demonstrated that the mean cell size of *Synechococcuss* new-born cells followed the predicted negative correlation with temperature suggested by TSR. Cyanobacteria cells became larger during the first period of light cycle due to the biomass accumulation induced by photosynthesis and became smaller during the dark cycle as a result of mother cells division (Palacio et al., 2020). It remains to be explained, though, the positive association between environmental temperature and the size of the most abundant *Synechococcus* group detected by flow cytometry in the shallow waters of KAUST Harbor (Supplementary Figure 9), which was sampled always at the same time (ca. 9:00 a.m. local time) (Sabbagh et al., 2020). One potential explanation would involve the high salinities found in the Red Sea, which can induce the internal accumulation of osmolytes for protection, resulting in larger cell volumes (Brown, 1976). In other organisms such as the anemone *Aiptasia* (Gegner et al., 2017) it has been shown that this strategy to cope with high salinities can also been advantageous for high temperatures. A significant difference was determined in G1 phase (bacterial growth) and S phase (DNA synthesis) duration of a freshwater *Synechococcus* strain cell cycle among different salinity conditions (0, 10, 20, and 30 of salinity), but G2 phase (preparation for bacterial cell division) duration did not vary. Mean cell sizes of this freshwater *Synechococcus* strain after acclimating to higher salinity became larger, and the doubling time was also longer at the highest salinity (Bemal and Anil, 2018). These *Synechococcus* cells were found to investing more energy in producing exopolysaccharides than on cell division, resulting in reduced growth rate but larger cell size (Bemal and Anil, 2018).

In conclusion, the assessed *Synechococcus* strain and their associated heterotrophic bacteria responded to experimental warming differently, without any clear, common pattern for heterotrophs, suggesting that there will be no universal response of bacterioplankton assemblages to global warming. Overall, our results were poorly described by the MTE and TSR. Within the assessed temperature range (24–34°C), which is typical for a substantial portion of the Red Sea (Raitsos et al., 2013; Chaidez et al., 2017), increasing temperature resulted in higher specific growth rate and cell size for *Synechococcus*, reaching their maximum at 33∘C, similar to other tropical and sup-tropical cyanobacteria strains that showed maximum growth rates at relatively higher temperatures (32–34∘C) (Pittera et al., 2014). However, heterotrophic bacterial specific growth rates did also increase with warming, cell sizes did not change in a coherent way. Our data suggest that temperatures equal or higher than 34∘C can already be detrimental for the cyanobacterium strain and one major heterotrophic bacteria player (*Muricauda*) in the RS9907 co-culture. The role of the three dominant heterotrophic bacteria in this cyanobacterial co-culture needs to be further analyzed and future metagenomic and metatranscriptomic studies may allow a deeper understanding on the metabolic interactions between autotrophic and heterotrophic bacteria in response to temperature.

## Data Availability Statement

The datasets presented in this study can be found in online repositories. The names of the repository/repositories and accession number(s) can be found in the article/Supplementary Material.

## Author Contributions

AL performed experiments A and C, analyzed data of all experiments, contributed in the experimental design of experiment C, and wrote the first draft of the manuscript. AP performed experiment A and contributed in the experimental design of experiment C. FG performed experiment B and contributed in editing the draft. GH contributed in editing the draft. MA collected time series data. ÁL-U designed experiment A. LA-S designed experiment A and contributed in editing the draft. P-YH supervised the study, contributed in editing the draft, and approved the final draft. XAGM designed experiment C, supervised the study, contributed in editing the draft, and approved the final draft. All authors contributed to the article and approved the submitted version.

## Conflict of Interest

The authors declare that the research was conducted in the absence of any commercial or financial relationships that could be construed as a potential conflict of interest.
