# Supplementary material for: Temperature Responses of Heterotrophic Bacteria in Co-culture With a Red Sea Synechococcus Strain
Source: Front Microbiol. 2021 May 10;12:612732. doi: 10.3389/fmicb.2021.612732 (PMC8141594; doi:10.3389/fmicb.2021.612732)
Supplement: Supplementary file 1 [file Table_1.pdf]

Supplementary Table 1: Details about experiments A, B, and C

Experiment	Tested temperatures	Duration and sampling interval	Initial abundance ± SD	Aim of the experiment	Data obtained from each experiment
**A**	4: 24, 28, 30 and 33˚C	13 days / daily	**Autotrophs:** 2.5 x 10^5 ± 205552<br><br>**Heterotrophs:** 8.3 x 10^4 ± 62882	Monitoring the dynamics of *Synechococcus*	- Carrying capacity of *Synechococcus* and *Muricauda*<br>- Variations in the abundance and mean cell size of all bacterial groups
**B**	4: 24, 28, 30, and 33˚C	4 days / no sampling (days 1-2) and every 2 hours (days 2-4)	**Autotrophs:** 2.8 x 10^6 ± 1567599<br><br>**Heterotrophs:** 8.2 x 10^6 ± 1258003	Documenting the short-term dynamics of *Synecochoccus* and heterotrophic bacteria	- Variations in the abundance and mean cell size of all bacterial groups
**C**	5: 24, 28, 30, 33 and 34˚C*	2 days / every 2 hours (day 1) and after 48 hours (day 2)	**Autotrophs:** 1 x 10^5 ± 6886<br><br>**Heterotrophs:** 5.8 x 10^4 ± 17687	Targeting the short-term dynamics of heterotrophic bacteria	- Specific growth rate, mean cell size, activation energy of all bacterial groups<br>- Mortality rate of *Paracoccus*<br>- Carrying capacity of *Paracoccus* and *Marinobacter*<br>- *16S rRNA* identification of heterotrophic groups distinguished by flow cytometry<br>- Variations in the abundance and mean cell size of all bacterial groups

* From experiment A and B we learnt that most of the bacterial groups were already stressed at 33˚C. As the study is more focused on experiment C, we acclimated the cultures at one more temperature higher than 33˚C but still within the natural temperature range of the Red Sea.

Supplementary Table 2: Mean contribution (%) of heterotrophic bacteria and RS9907 *Synechococcus* to total cell abundance counted by flow cyometry at the different temperatures of experiment C after 48 h (the time of DNA samples collection).

**Temperature (˚C)**	**Heterotrophic bacteria (%) ± SD**	***Synechococcus* (%)**
24	91.3 ± 0.01	8.7
28	83.6± 0.00	16.4
30	82.8± 0.01	17.2
33	78.2± 0.01	21.8
34	71.2± 0.02	28.8

Supplementary Table 3: Mean cell sizes of *Synechococcus* RS9907 and its associated heterotrophic bacteria at the three experiments.

Experiment	Genus	Biovolume (μm^3) ±SD				
		**24˚C**	**28˚C**	**30˚C**	**33˚C**	**34C**
Experiment **A**	***Synechococcus***	0.42 ± 0.03	0.54 ± 0.01	0.64 ± 0.01	0.72 ± 0.02	NA
	Paracoccus	0.17 ± 0.01	0.18 ± 0.01	0.17 ± 0.01	0.19 ± 0.01	NA
	Marinobacter	0.14 ± 0.01	0.15 ± 0.00	0.14 ± 0.01	0.15 ± 0.01	NA
	Muricauda	0.12 ± 0.01	0.12 ± 0.01	0.11 ± 0.01	0.11 ± 0.01	NA
Experiment **B**	***Synechococcus***	0.57 ± 0.01	0.61 ± 0.02	0.67 ± 0.01	0.79 ± 0.01	NA
	Paracoccus	0.20 ± 0.01	0.21 ± 0.01	0.21 ± 0.01	0.20 ± 0.00	NA
	Marinobacter	0.16 ± 0.00	0.16 ± 0.01	0.16 ± 0.00	0.15 ± 0.01	NA
	Muricauda	0.12 ± 0.00	0.12 ± 0.00	0.12 ± 0.00	0.12 ± 0.01	NA
Experiment **C**	***Synechococcus***	0.39 ± 0.01	0.47 ± 0.01	0.60 ± 0.02	0.53 ± 0.01	0.56±0.03
	Paracoccus	0.19 ± 0.00	0.19 ± 0.00	0.20 ± 0.00	0.19 ± 0.00	0.20±0.01
	Marinobacter	0.15 ± 0.01	0.14 ± 0.01	0.15 ± 0.00	0.15 ± 0.01	0.15±0.00
	Muricauda	0.12 ± 0.00	0.12 ± 0.00	0.12 ± 0.01	0.12 ± 0.01	0.12±0.01

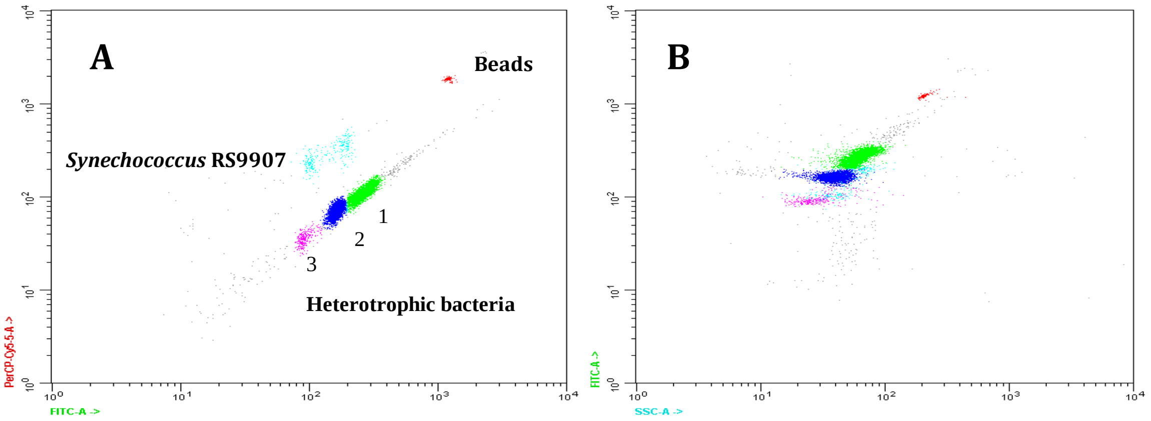

Supplementary Figure 1: Example of cytogram (experiment C, at 24°C) showing *Synechococcus* RS9907 and 3 heterotrophic bacteria populations (numbered by decreasing values of PerCP-Cy5-5, FITC and SSC). The *Synechococcus* cluster is easily distinguished in A by their high red fluorescence signal (PerCP-Cy5-5) due to chlorophyll *a* vs. green fluorescence (FITC) after nucleic acid staining. B shows that the 3 heterotrophic bacterial groups are characterized by different values of nucleic acid content (FITC signal) and cell size using (right angle side scatter or SSCsignal as a proxy).

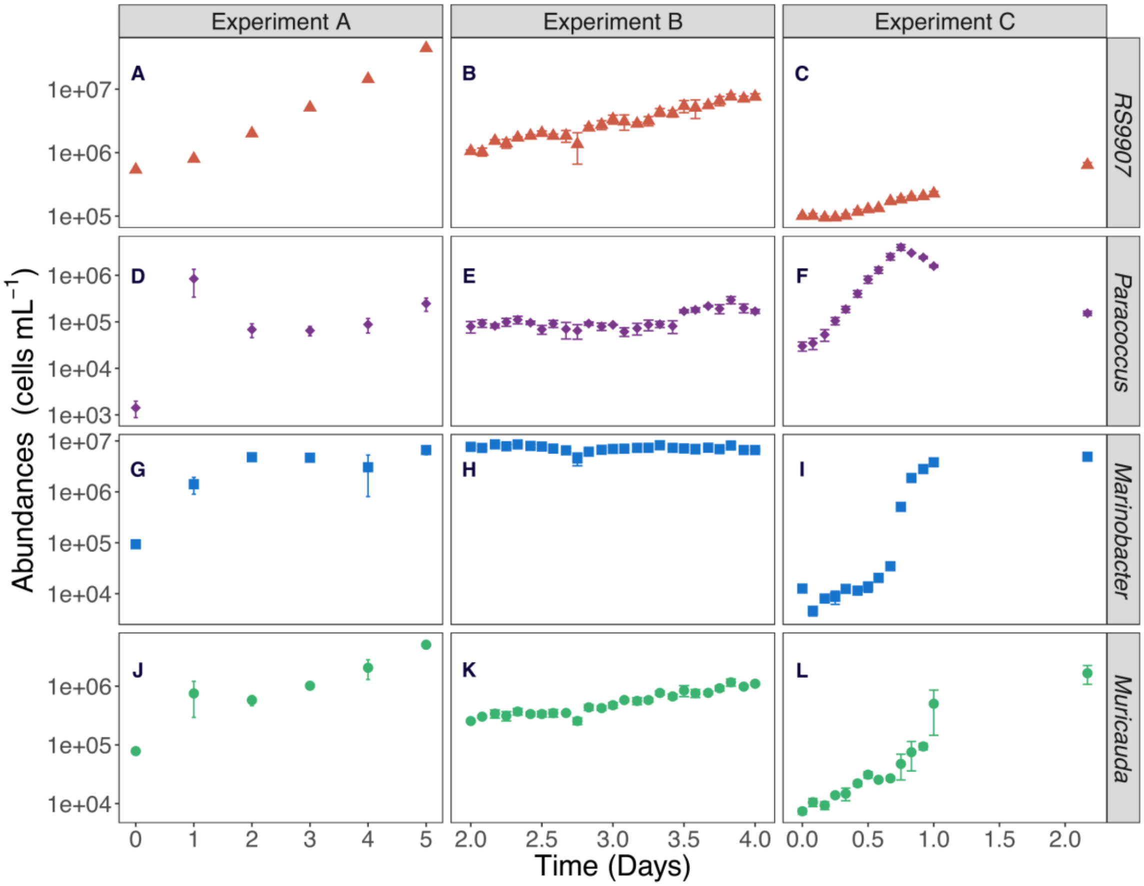

Supplementary Figure 2: Variations in the mean abundance of *Synechococcus* RS9907 (A-C) and the 3 heterotrophic bacteria (*Paracoccus* D-F, *Marinobacter* G-I, *Muricauda* J-L) at at 24˚C in the three experiments. Error bars represent standard deviations of 4 replicates in experiment A triplicates in experiments B and C.

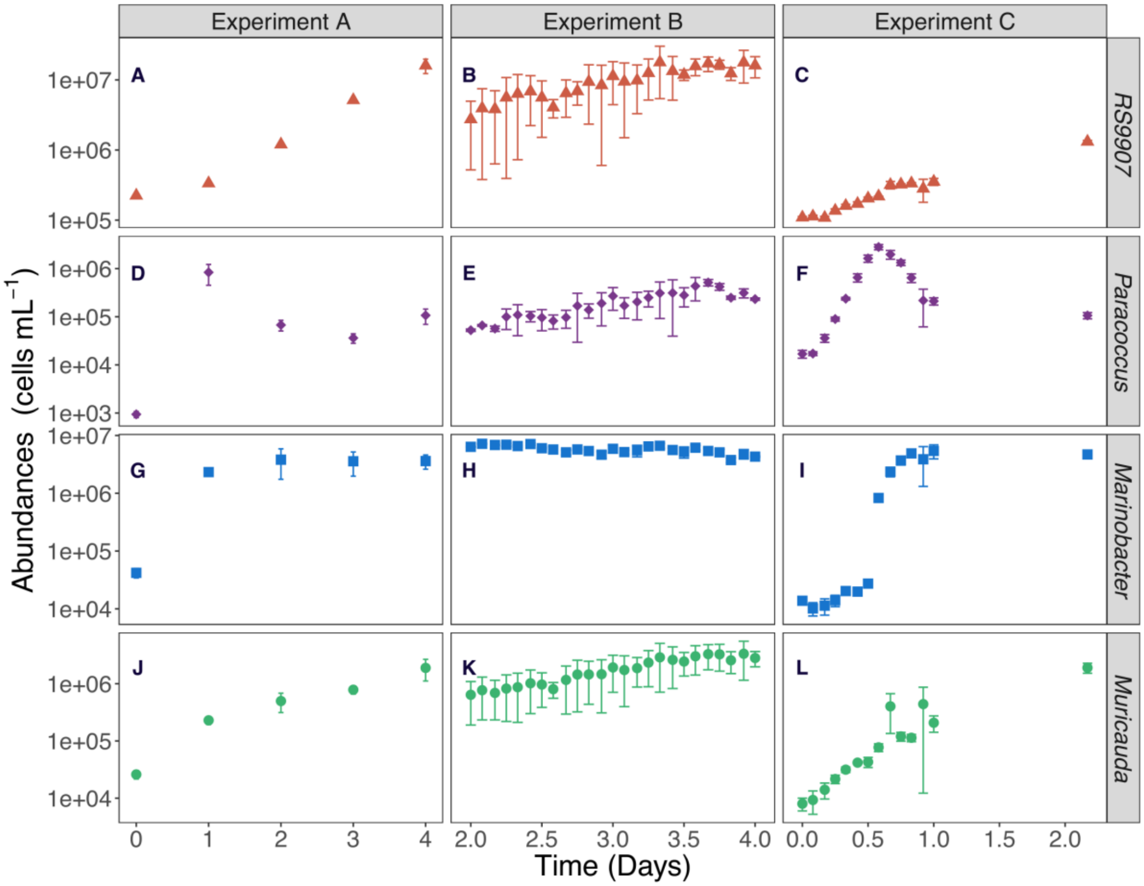

Supplementary Figure 3: Variations in the mean abundance of *Synechococcus* RS9907 (A-C) and the 3 heterotrophic bacteria (*Paracoccus* D-F, *Marinobacter* G-I, *Muricauda* J-L) at 28˚C in the three experiments. Error bars represent standard deviations of 4 replicates in experiment A triplicates in experiments B and C.

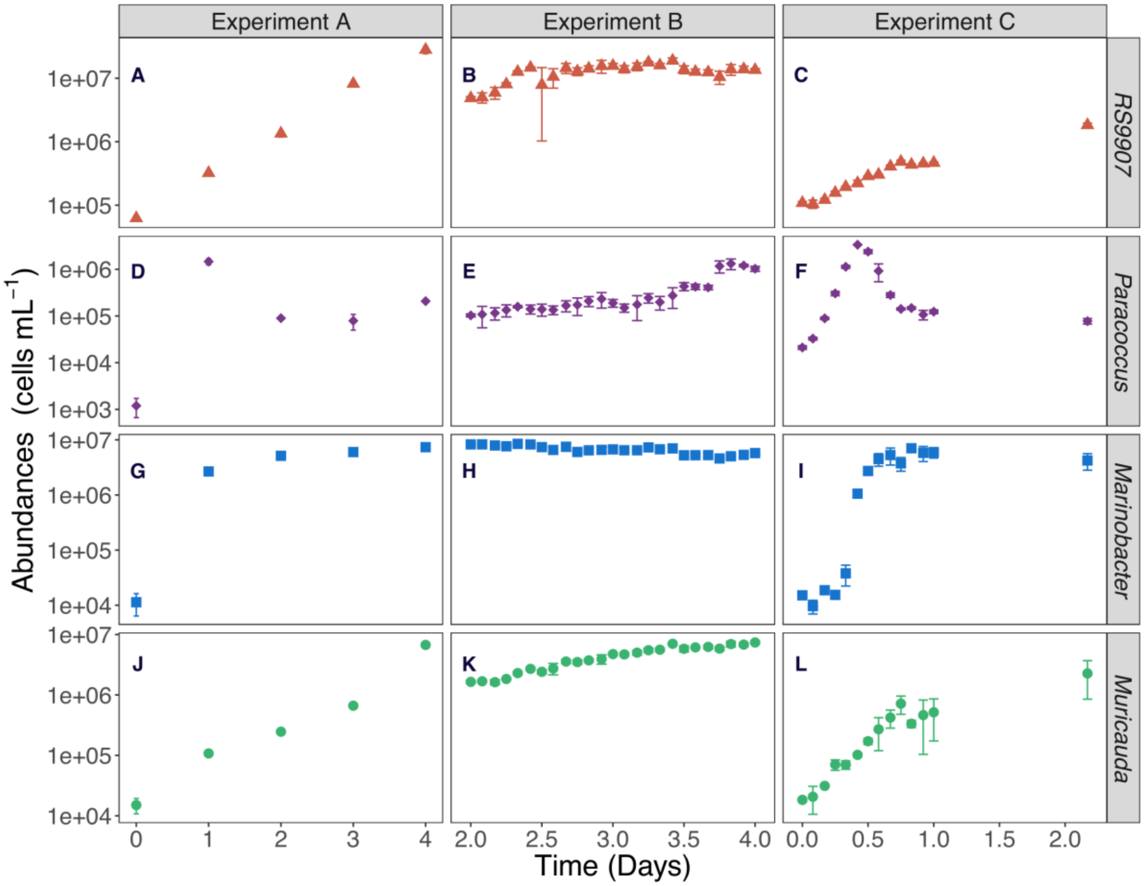

Supplementary Figure 4: Variations in the mean abundance of *Synechococcus* RS9907 (A-C) and the 3 heterotrophic bacteria (*Paracoccus* D-F, *Marinobacter* G-I, *Muricauda* J-L) at 33˚C in the three experiments. Error bars represent standard deviations of 4 replicates in experiment A triplicates in experiments B and C.

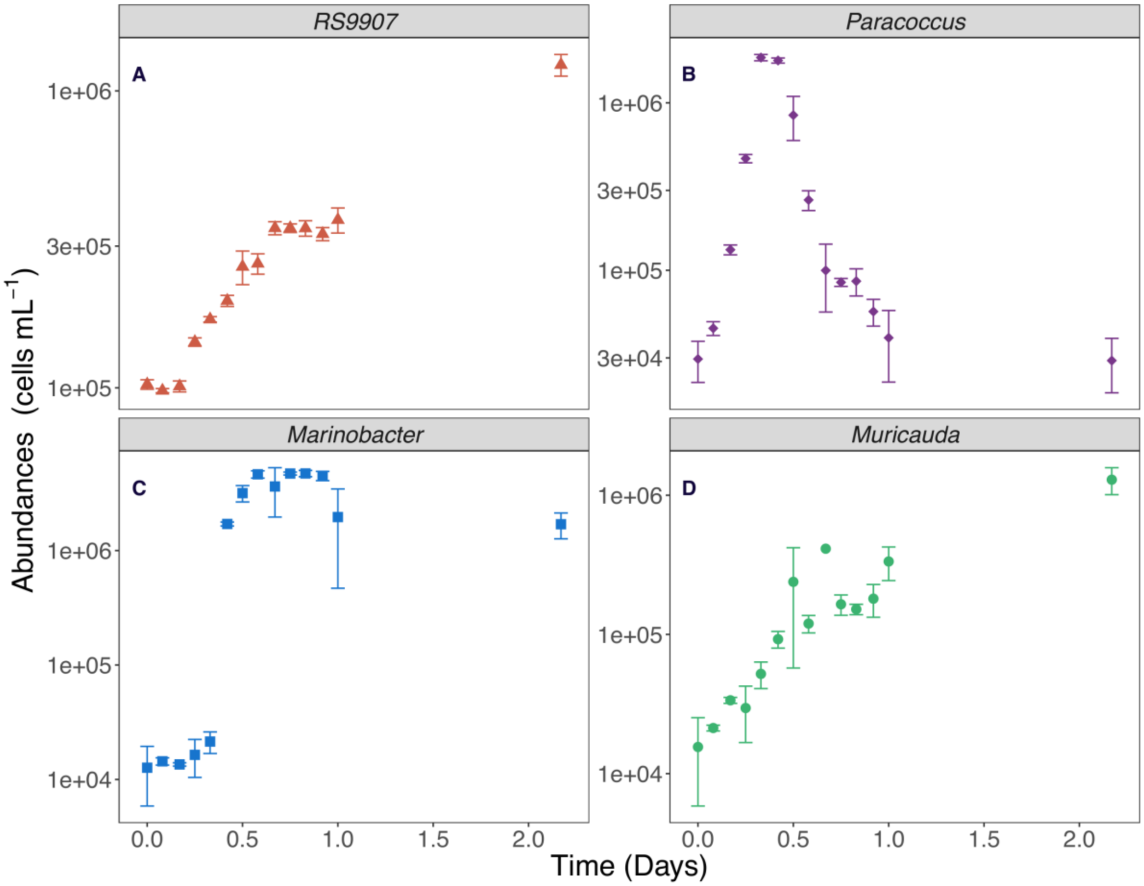

Supplementary Figure 5: Variations in the abundance of *Synechococcus* RS9907 (A), *Paracoccus* (B), *Marinobacter* (C) and *Muricauda* (D) at 34˚C in experiment C. Error bars represent standard deviations of triplicates.

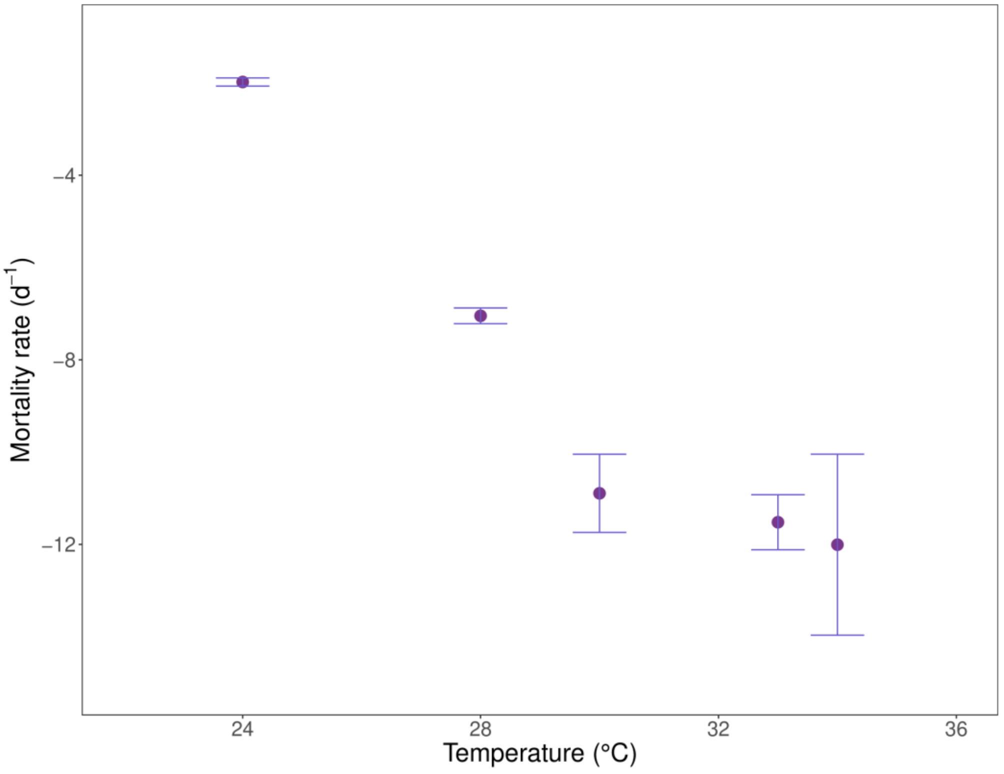

Supplementary Figure 6: Estimated mortality rates of *Paracoccus* at 5 different temperatures of experiment C. Error bars represent standard deviations of triplicates.

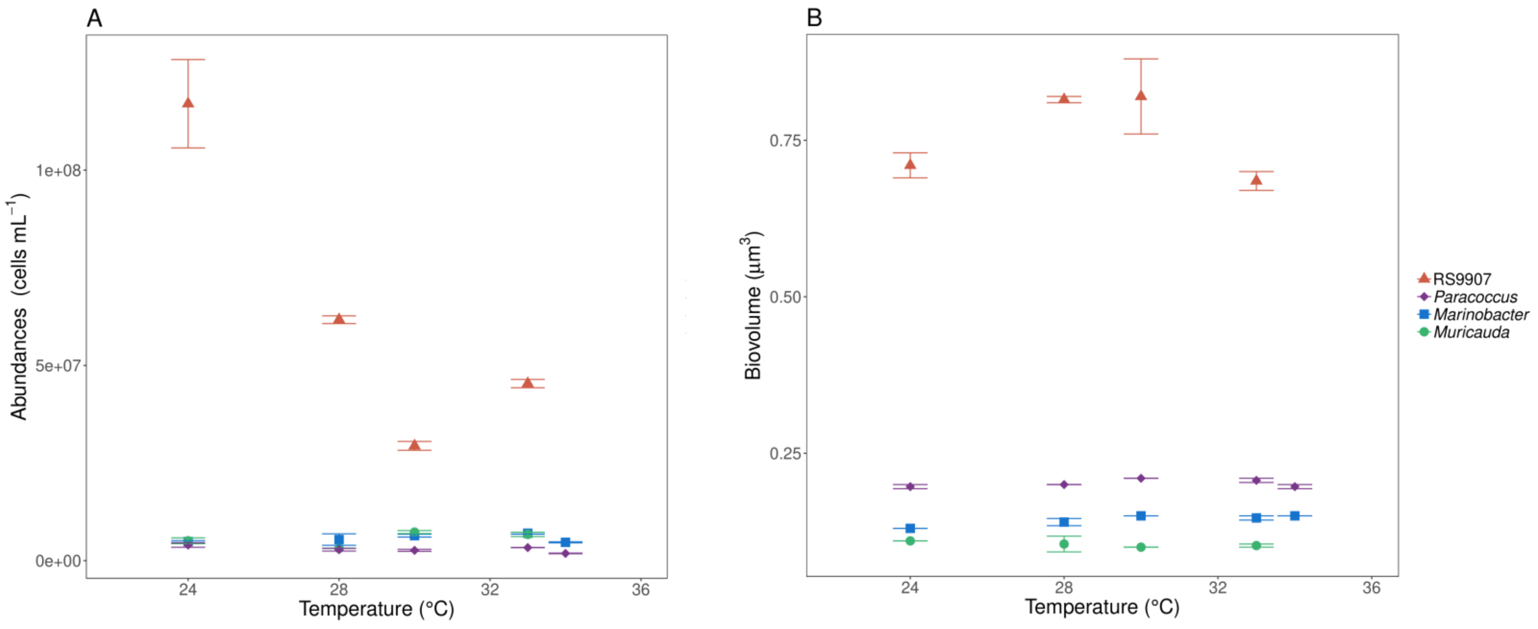

Supplementary Figure 7: Mean carrying capacity (i.e. maximum abundance, A) of *Synechococcus* RS9907 and their associated heterotrophic bacteria and their corresponding cell size (B) at maximum abundance. Error bars represent standard deviations.

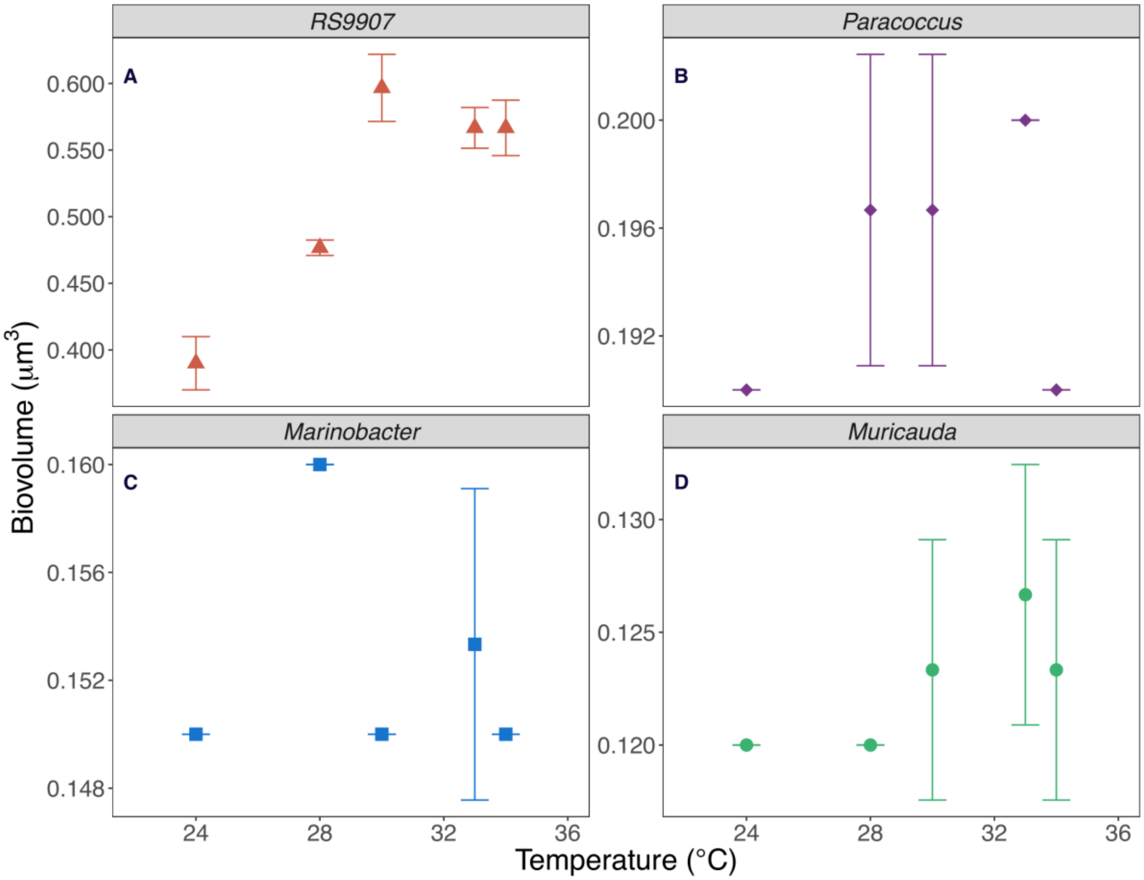

Supplementary Figure 8: Mean cell sizes of *Synechococcus* RS9907 (A), *Paracoccus* (B), *Marinobacter* (C) and *Muricauda* (D) vs. temperature during the exponential growth phase of experiment C. Error bars represent standard deviations.

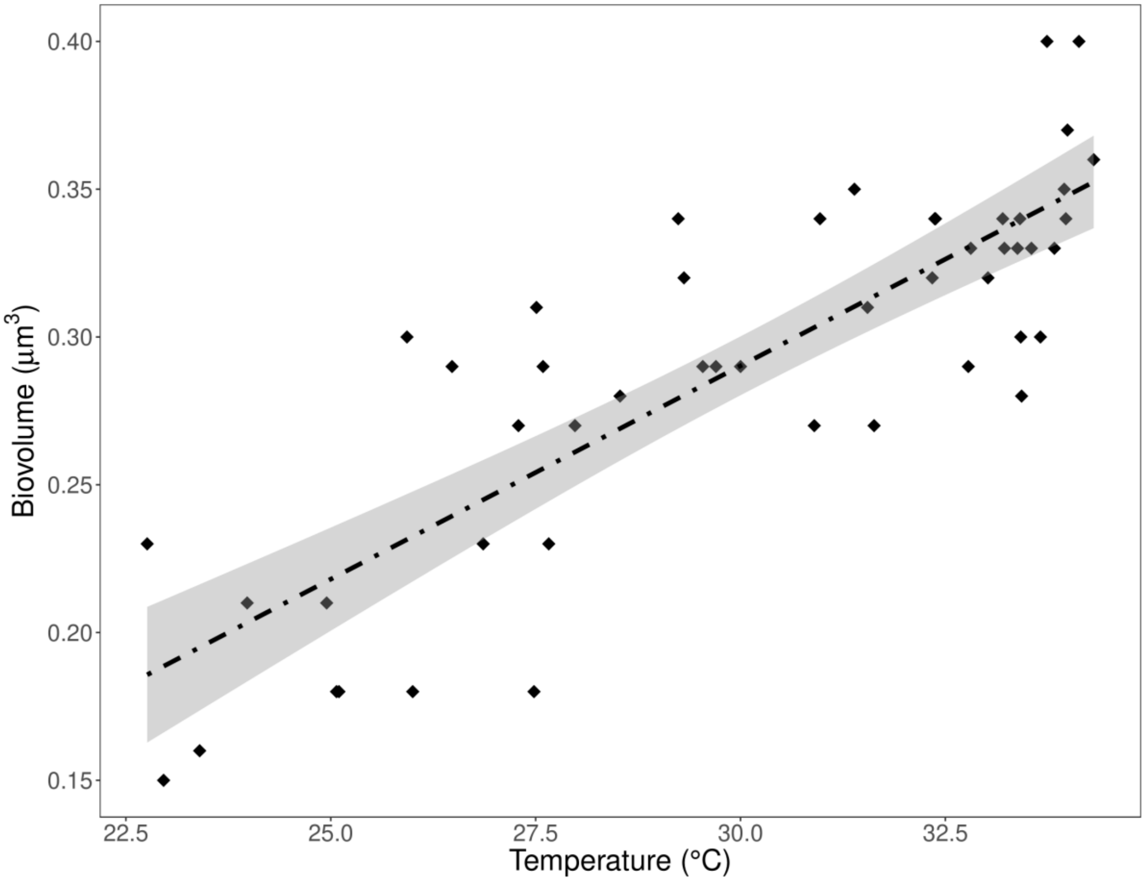

Supplementary Figure 9: Relationship between mean cell size and ambient temperature of a natural population of *Synechococcus* found year-round in shallow waters of the central Red Sea. Each dot corresponds to a weekly sample conducted in 2016. The fitted line represents the linear regression (r^2=0.68, p-value < 0.00001, n=49).
